# Endoscopic Treatment of Symptomatic Foot and Ankle Bone Cyst with 3D Printing Application

**DOI:** 10.1155/2020/8323658

**Published:** 2020-12-26

**Authors:** Changgui Zhang, Jin Cao, Hongli Zhu, Huaquan Fan, Liu Yang, Xiaojun Duan

**Affiliations:** Center for Joint Surgery, Southwest Hospital, Third Military Medical University (Army Medical University), Chongqing 400038, China

## Abstract

**Objective:**

To study the efficacy of arthroscopy for treating symptomatic bone cysts of the foot and ankle through the follow-up of patients and to further explore the application value of 3D printing technology in this treatment.

**Methods:**

Twenty-one patients with symptomatic bone cysts in the foot and ankle who underwent arthroscopic surgery in our Center from March 2010 to December 2018 were enrolled, including 11 in the experimental group and 10 in the control group. For the control group, C-arm fluoroscopy was used intraoperatively to confirm the positioning of the cysts; for the experimental group, a 3D model of the lesion tissue and the 3D-printed individualized guides were prepared to assist the positioning of the cysts. Debridement of the lesion tissues was conducted under an arthroscope. Regular follow-ups were conducted. The time of establishing arthroscopic approaches and the times of intraoperative fluoroscopy between the two groups were compared. Significance was determined as *P* < 0.05.

**Results:**

The postoperative pathology of the patients confirmed the diagnosis. No significant perioperative complications were observed in either group, and no recurrence of bone cysts was seen at the last follow-up. The VAS scores and AOFAS scores of the two groups at the last follow-up were significantly improved compared with the preoperative data, but there was no statistical difference between the two groups. All surgeries were performed by the same senior surgeon. The time taken to establish the arthroscopic approaches between the two groups was statistically significant (*P* < 0.001), and the times of intraoperative fluoroscopy required to establish the approach were also statistically significant (*P* < 0.001). The intraoperative bleeding between the two groups was statistically significant (*P* < 0.01). There was 1 case in each group whose postoperative CT showed insufficient bone grafting, but no increase in cavity volume was observed during the follow-up.

**Conclusion:**

With the assistance of the 3D printing technology for treating symptomatic bone cysts of the ankle and foot, the surgeon can design the operation preoperatively and perform the rehearsal, which would make it easier to establish the arthroscopic approach, better understand the anatomy, and make the operation smoother. This trial is registered with http://www.clinicaltrials.govNCT03152916.

## 1. Introduction

Bone cysts of the extremities are tumor-like lesions, which mainly occur in the femoral neck, the proximal femur, and the proximal humerus, while those occurring in the foot and ankle are relatively rare. For example, the incidence of talar bone cysts accounts for 0.003% of the bone tumor, and they are commonly diagnosed as simple bone cysts, aneurysmal bone cysts, intraosseous ganglion, etc. [1–8]. The etiology and mechanism of bone cysts have not been fully elucidated. Some studies have suggested that talar bone cysts were related to the subchondral bone fissures and the increased pressure of fluid in the subchondral bone. Fluid pressure and osteocyte apoptosis might lead to the growth of cysts via mechanically regulated bone adaptation [9]. Bone cysts generally have no obvious symptoms. Most of its patients seek treatment due to pathological fractures that lead to pain, swelling, and dysfunction. They can be diagnosed by radiographic examination. However, in the clinic, some patients have bone cysts that demonstrate progressive development in a short period of time, and bone destruction is gradually aggravated. If not treated in time, these symptomatic bone cysts can easily lead to pathological fractures. Recent literature has also shown that the destruction of subchondral bone is also an important cause of cartilage damage, and its development can be followed by osteoarthritis [10, 11]. Thus, patients with poor conservative treatment results should consider surgery.

Arthroscopy has the advantages of smaller trauma and clearer vision under the arthroscope, which make it suitable for intra-articular surgery. Besides, the incision is good-looking, and the postoperative joint function recovery is fast. Hence, arthroscopy develops rapidly in recent years and is well received by patients. It has been widely used in foot and ankle surgeries, such as talar osteochondral injury, bony impingement syndrome, ankle joint arthrodesis, ankle joint tuberculosis, and even some benign tumors of the ankle joint [12, 13]. With the continuous advancement of endoscopic techniques, endoscopic treatment of bone cysts has also been reported in some literature recently [1, 4–8, 14, 15]. However, the literature on endoscopic treatment of ankle bone cysts is still very rare, and the validity of this new technology remains controversial. Three-dimensional (3D) printing technology is a hot new technology in orthopedics [16–22], which is especially suitable for individualized precision treatment. We have utilized the 3D printing technology in fields such as ankle joint arthrodesis and subtalar joint arthrodesis and have obtained significant results [17, 18]. In this study, we followed the patients with symptomatic bone cysts of the foot and ankle that were treated with arthroscopic technique in recent years to estimate its application value and to further explore the specific application value of the 3D printing technology, providing a basis for its future promotion.

## 2. Materials and Methods

### 2.1. Participants

Patients with symptomatic bone cysts in the foot and ankle and who received surgical treatment in our Center from March 2010 to December 2018 were followed. Indications for surgery are the following: (1) after 6 months of conservative treatment, there were still symptoms of bone cysts; (2) 3D computed tomography (CT) examination indicated that the bone destruction volume > 1 cm^3^. Case inclusion criteria are the following: (1) patients who received arthroscopic treatment for symptomatic bone cysts of the ankle and foot, (2) complete preoperative imaging data, and (3) bone cysts of similar size (1 cm^3^-3 cm^3^). Exclusion criteria are the following: (1) history of previous foot and ankle surgery; (2) history of ankle fracture; (3) ankle joint deformity, abnormal alignment of the lower extremity; (4) severe osteoarthritis of the ankle or subtalar joint; (5) pathological findings suggesting malignant bone tumors; (6) recurrence of bone cysts after previous treatment; and (7) history of mental illness. Twenty-one patients were followed. All cases were unilateral. (See Table 1 for details.)

All patients were informed before surgery that there was a risk of recurrence of bone cysts due to incomplete removal of the lesions by arthroscopic techniques. In addition, if the articular cartilage showed obvious damage, the second-stage treatment of cartilage would be required. All surgeries in this study were performed by the same senior surgeon. The study was approved by the hospital's ethics committee, and all patients signed the informed consent form and agreed to be enrolled in the study.

### 2.2. Preoperative Individualized Application of the 3D Printing Technology

**Figure 1:**
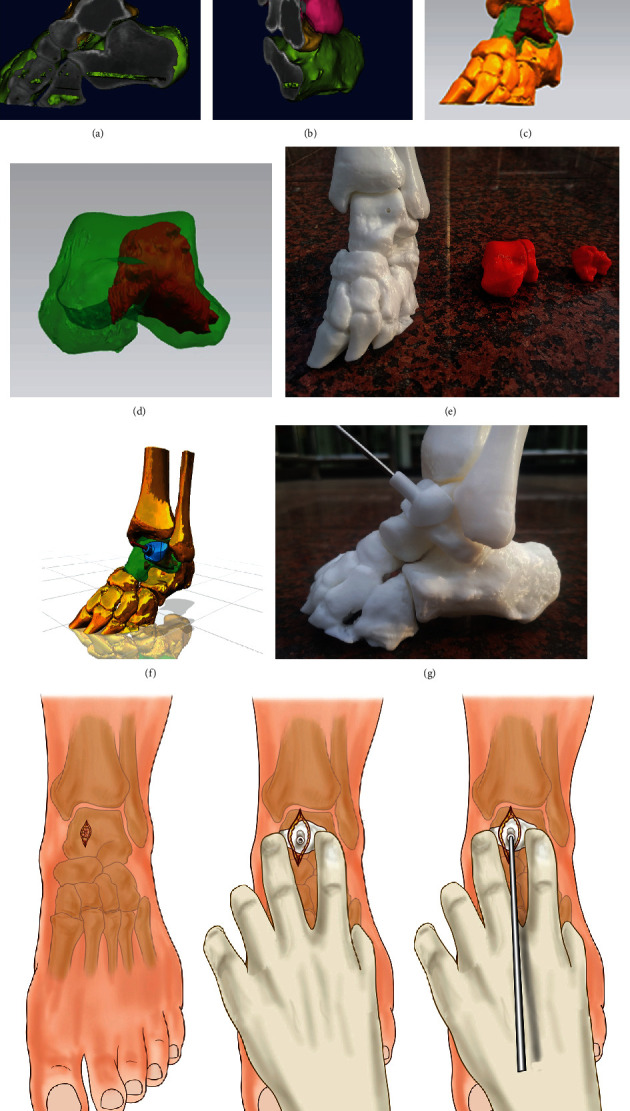
Preoperative preparation of 3D-printed lesion models and individualized guide system. (a, b) Talar lesions established based on the 3D CT images before surgery; (c, d) the software was used to distinguish lesions from healthy tissues; (e) 3D-printed lesion model that could be used for surgical planning and rehearsal; (f, g) design and preparation of individualized guides to assist in determining the position of the Kirschner wire. (h) The location of the lesion was entered through the incision, and the bony structure was confirmed; (i) the anatomical position was determined through the bony structure, and the cyst position was located with the guide; (j) the guide was used to assist the accurate positioning of the Kirschner wire.

A 1 : 1 proportional model of the lesion and individualized surgical guide were prepared for the experimental group. The 3D CT thin-layer scan (Siemens, Germany) of the ankle bone cyst was routinely performed, and the CT scan slice thickness was 1 mm. The Digital Imaging and Communications in Medicine (DICM) data was extracted and imported into the Model Intestinal Microflora in Computer Simulation (MIMICS) software to reconstruct the 3D data of the bone cyst and its surrounding tissues. The MIMICS-reconstructed data was imported into the 3D design software SIEMENS NX (Siemens PLM Software, Germany) to design the guide system for arthroscopy. The individualized guides were designed according to the anatomical landmarks to perfectly match with the bone surface, and the center of the guide contained guide holes for the Kirschner wire to drill (Figure 1). The data of the designed guides were converted into STL format and imported into a 3D printer (Model: UP BOX, Tiertime, China) for printing. Polylactic acid (Tiertime, China) was used as a raw material to prepare the individualized guides (Figure 1), and lesion models were prepared. When preparing the lesion models, different colors could be used to distinguish the lesion from the surrounding healthy tissues, which was beneficial to improving surgical planning. The individualized guides were sealed and sterilized with ethylene oxide for intraoperative use.

### 2.3. Operative Techniques

Nerve block anesthesia or epidural anesthesia were conducted for all patients. Thigh tourniquets were applied after successful anesthesia. Appropriate position was chosen according to the location of the bone cyst. If it was a distal tibial bone cyst, the patient could use the routine supine position; if it was a calcaneal bone cyst, the lateral position should be used. In cases with the talar bone cyst, the supine position was usually selected. In order to facilitate the operation, the hip on the affected side was moderately elevated to maintain the neutral position of the lower limbs. After routine sterilization and draping, the tourniquet pressure was set to 300 mmHg.

**Figure 2:**
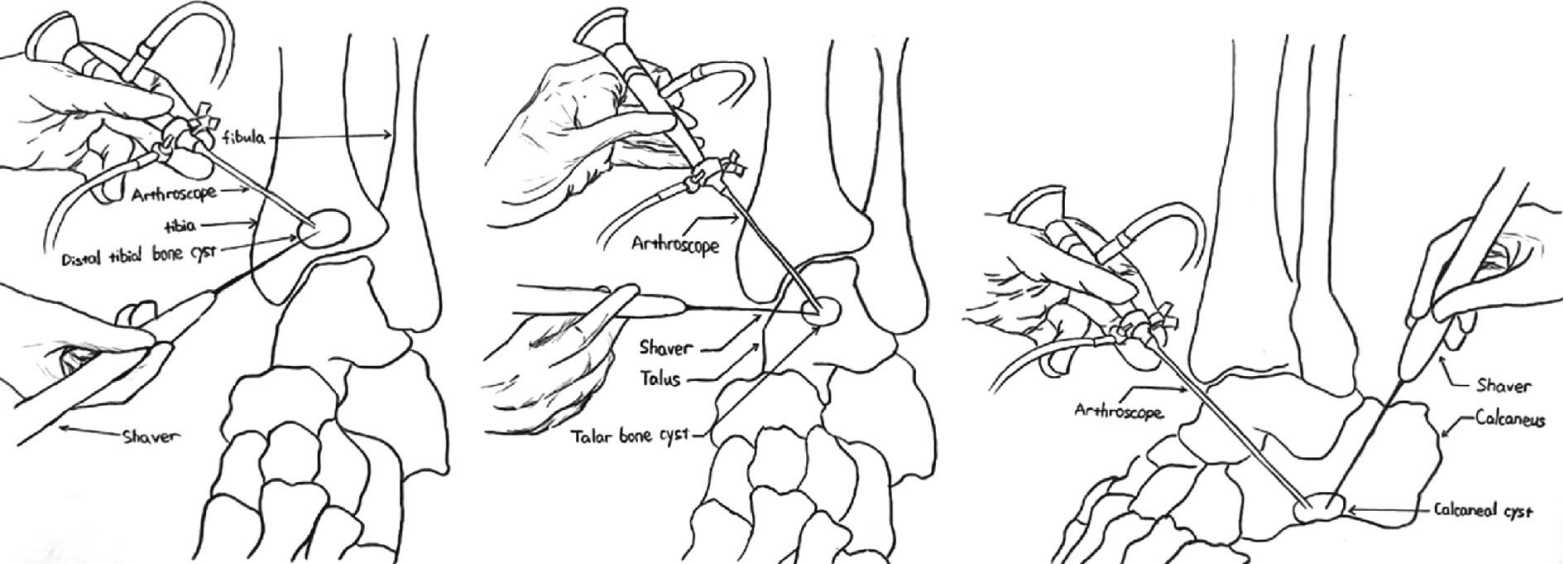
Arthroscopically assisted treatment of bone cysts via different approaches.

The talar bone cyst was taken as an example to elucidate the surgical procedures. After accurate positioning, 10-20 mL saline was injected into the ankle joint cavity; the anteromedial and anterolateral ankle approaches were routinely established, and the 30-degree arthroscopy (Smith & Nephew) was placed into the joint cavity through these approaches. Careful exploration of the joint cavity was conducted to observe whether there was synovial hyperplasia or corresponding articular cartilage injury; lesions would be treated if detected. After the exploration and debridement, the traction device of the ankle joint was removed, and the medial or lateral incision was moderately enlarged by about 3 cm. The tendon tissue was pulled outward for the drill of the Kirschner wire to drill. Care should be taken not to damage the nerves, blood vessels, ligaments, etc., in this process. For the control group, the surgeon did not use the 3D-printed guide and drilled the Kirschner wire relying on C-arm fluoroscopy. For the experimental group, besides previous experience, the surgeon was assisted by the 3D-printed lesion model and guide for the accurate positioning and drilling of the 2.0 mm Kirschner wire. Attention should be paid to the drilling of the Kirschner wire to avoid the articular cartilage. Then, C-arm fluoroscopy was applied to confirm whether the Kirschner wire had entered the lesion and whether its position met with the preoperative planning; otherwise, it needed to be redrilled. A 4.5 mm drill was drilled into the intraosseous lesion along the Kirschner wire to establish the arthroscopic approach. During this process, there might be a sudden sense of falling out, and at the same time, the fluid of the cyst was often seen. These would confirm the entrance into the cyst. When the cystic fluid was not obvious, a 2.7 mm arthroscope could be used to enter the cavity for probing and adjusting the direction of operation. If the operation under the arthroscope was difficult, the diameter of the approach could be moderately enlarged. The bone lesions were explored (Figure 2). The tissue morphology of the cyst wall and the nature of the cyst fluid under an arthroscope were observed, and the pathological examination of the lesion tissue was conducted. The cyst wall was scraped with a curette repeatedly to ensure the complete removal of the lesion tissue. A burr was used to drill the sclerotic rim of the wall. Arthrocare radiofrequency ablation of the cystic wall was performed to prevent recurrence of the cystic wall lesions. The cyst wall was drilled using a 1.2 mm Kirschner wire for the infiltration of bone marrow to promote bony union. Finally, the bone defect was impacted tightly with autologous cancellous bone harvested from the iliac crest, allograft cancellous bone, or bone cement. C-arm fluoroscopy was applied to confirm the complete debridement of the cyst and satisfactory filling of the bone defect. A drain was placed into the joint after irrigation. The portals were closed with interrupted sutures. The plaster cast was used to fix the ankle joint to the functional position.

For bone cysts in different locations, we used different methods. If the bone cyst was in the talus, we chose the anterior approach for arthroscopic removal; if it was in the tibia, we performed arthroscopic removal on the anterior part of the tibia; if it was in the calcaneus, removal was conducted on the anterior part of the calcaneus. (Drawn by Changgui Zhang.)

After the anesthetic effect wore off, the patients should start toe activity and perform functional exercises under instruction. The drainage tube was removed 24 hours postoperatively, and the antibiotics were intravenously infused for 48 hours. The suture was removed 2 weeks postoperatively, and partial weight-bearing walking was started from 4 weeks on. Perioperative complications were recorded. Regular follow-ups and corresponding imaging examinations were performed.

### 2.4. Indicators

The time of surgery was recorded, including the time taken to establish the arthroscopic approach and the times of C-arm fluoroscopy used during the period. The time of establishing arthroscopic approaches was the time from the beginning of the arthroscopic approach establishment to the finish. For the times of C-arm fluoroscopy, it was defined as the times of fluoroscopy applied from the beginning of surgery to its finish. The intraoperative blood loss was recorded. Preoperative and postoperative VAS scores and AOFAS scores were recorded [23, 24]. All patients were followed at 1 month, half a year, and one year after operation; the results of radiographic examinations before and after operation were compared. If the postoperative radiographs were insufficient to confirm the condition, CT examination would be required. At the last follow-up, an ankle joint 3D CT reconstruction was performed to confirm the union of the bone cyst and whether there was a cyst recurrence.

### 2.5. Statistical Analysis

The measuring index was statistically analyzed by SPSS 22.0 using the mean ± standard deviation, and the indexes of the experimental group and the control group were compared before and after the operation. If the data conformed to the normal distribution, the *t*-test would be used; if not, the rank sum test would be used. The enumeration data was processed by the chi-squared test. *P* < 0.05 was considered statistically significant.

## 3. Results

**Figure 3:**
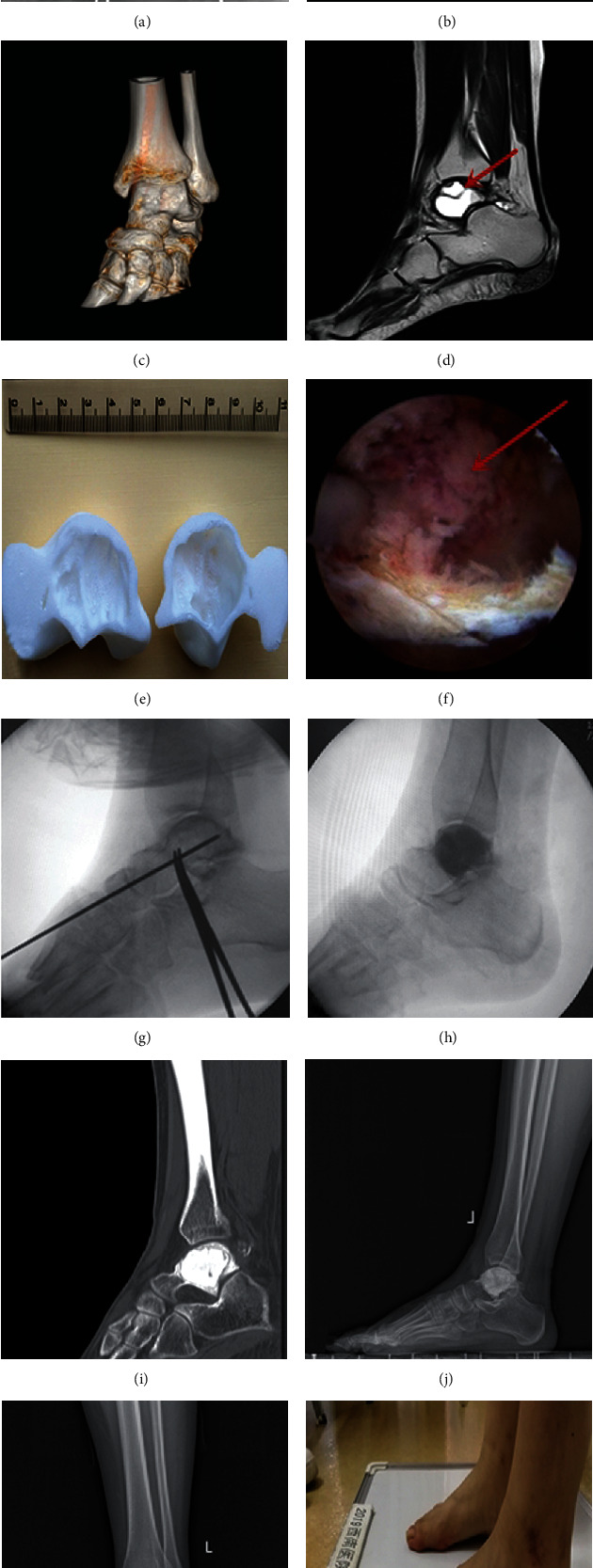
Typical case 1 in the experimental group. Female, 15 years old, admitted to the hospital because of left ankle pain for 1 year. Preoperative diagnosis: pathological fracture secondary to the left talar bone cyst. (a–d) Postoperative examinations; the arrows indicated the location of the bone cyst. (e) 3D-printed lesion model; (f) arthroscopic observation of cyst lesion, and the red arrow showed the bone cyst; (g) the Kirschner wire was drilled through the anterior arthroscopic approach with the assistance of individualized guides, and C-arm fluoroscopy was used to confirm that the Kirschner wire had accurately entered the talar cyst site; (h) fluoroscopy was used to confirm the optimal filling of the bone cement; (i) no recurrence of bone cysts was seen in the 3D CT reconstruction after 18 months; (j, k) no recurrence of bone cysts was seen in the radiographs at the last follow-up after 18 months; (l) the left ankle symptoms were relieved at the last follow-up, and the AOFAS score was 94 points.

All 21 patients were followed for 40 ± 22 (12-72) months. The final pathological types were 15 cases of simple bone cyst, 5 cases of aneurysmal bone cyst, and 1 case of intraosseous ganglion. No patients had perioperative complications such as infection, neurovascular injury, lower extremity venous thrombosis, or poor wound healing. The VAS score and AOFAS score at the last follow-up of the two groups were significantly improved compared with preoperative ones, but there was no statistical difference between the two groups (Table 2). There was a statistically significant difference in the time taken to establish an arthroscopic approach between the two groups during surgery (*P* < 0.001), and the intraoperative fluoroscopy times required to establish the approach was statistically significant (*P* < 0.001). The intraoperative bleeding between the two groups was statistically significant (*P* < 0.01). At the last follow-up, the radiographs and 3D CT reconstruction of the two groups confirmed the union of the bone cyst with no recurrence (Figure 3). However, there was 1 case in each group whose postoperative radiograph showed insufficient bone grafting at the first follow-up, and the following CT confirmed the insufficiency. However, no obvious increase in the defection area was observed via CT at the last follow-up, and the patient did not complain of pain and discomfort, so no special treatment was performed.

## 4. Discussion

Bone cysts are common in the clinic, but they are rarely seen in the foot and ankle. The etiology and mechanism of bone cysts have not been fully elucidated. There may be a unique mechanism for the formation of bone cysts in the ankle joint. Reilingh et al. studied 66 cases of fresh talar specimens and suggested that talar bone cysts were associated with the subchondral bone fissures and the increased fluid pressure; talar bone cysts were closely related to the cartilage injuries [9]. Due to persistent destruction, the bone cyst at the joint site is prone to lead to pathological fracture or cause osteoarthritis secondary to the cartilage defect [10, 11]. Therefore, surgical treatment should be considered for bone cysts in joints where symptoms persist. Since the local pain and joint function limitation in patients with bone cysts in the foot and ankle are often not particularly serious, if there are major surgical trauma or postoperative complications, the patient satisfaction rate will be greatly reduced. Therefore, surgeons have been exploring better minimally invasive techniques. In the treatment of talar bone cysts, traditional surgery generally involves osteotomy in order to clearly show the lesions [25, 26], but such surgery is more traumatic with prolonged postoperative recovery and may even lead to postoperative ankle joint function limitation. In recent years, with the improvement of arthroscopic equipment and the increasing skill of the technique, reports on arthroscopic treatment of the bone cysts in the foot and ankle have been published [3–5, 7, 11, 15, 27]. For example, Otsuka et al. debrided the calcaneal aneurysmal bone cyst with arthroscopy and achieved good results [3]. Ogut et al. reported treatment of posterior arthroscopy for the talar bone cyst and showed satisfactory outcomes at the short-term follow-up point [7]. This technique is especially suitable for bone cysts combined with other structural abnormalities at the posterior malleolus, such as tenosynovitis of flexor pollicis longus, symptomatic triquetral bone, and villonodular synovitis. Zhu et al. reported an anterior arthroscopic treatment for 7 cases of talar bone cysts, which also achieved satisfactory results in the short term [5]. In this study, the number of cases has been increased to 21; as for the location of cysts, in addition to the talus, distal tibia and calcaneus were also included. The short-term efficacy of arthroscopic treatment was quite satisfactory. This technique could avoid medial malleolar osteotomy and articular cartilage damage. It has the advantages of relatively safe, less traumatic, and quicker recovery. An arthroscope can be used to more intuitively observe the shape of the lesion; pathological examination of the tissue obtained during surgery can be used to further confirm the diagnosis [28–30]. Of course, there are also deficiencies in the application of this technique, such as a learning curve for surgeons, insufficient lesion scraping, and bone grafting.

Establishing an ideal arthroscopic approach is a critical step in achieving good surgical results. An ideal approach should meet the following requirements: (1) avoiding damage to important structures and close to the lesion tissue for easy operation and (2) providing clear vision to facilitate complete removal of the lesion. In the past, the surgeon relied on local anatomy and previous experience to drill the Kirschner wire for preliminary positioning. Then, C-arm fluoroscopy was used to confirm whether the position of the Kirschner wire was appropriate. In practice, this may cause repeated fluoroscopy for adjusting the position of the Kirschner wire, which would prolong the operation time and increases the intraoperative radiation. In recent years, 3D printing technology has been gaining popularity in orthopedics and has brought new hopes to solve the above problem. We first perform 3D reconstruction and analysis of the lesion, design the 3D-printed guide, and then utilize the 3D printing technology to print out the lesion models to comprehensively evaluate the lesions, observe the relationship between the lesions and the adjacent tissues, design a more scientific surgical plan and perform preoperative surgical rehearsals to help reduce the damage of the nerves and blood vessels, shorten the operation time, reduce intraoperative bleeding, and achieve better surgical results. It has been proved to be a safe and reliable new technology, which is especially suitable for individualized surgical treatment of difficult cases. Besides, the lesion model is particularly useful for communication between surgeons and patients, so that the patients can understand the disease and surgical content more clearly, which is helpful for increasing the patient satisfaction rate [31–36]. We used polylactic acid polymer materials for the 3D printing model and positioning guide plate, and the cost of materials was about RMB 300 yuan. We hope that with the development of technology, we can further reduce the time and cost. Bone graft after lesion debridement is a controversial topic. Some scholars advocated that bone graft should not be performed [2]. In our opinion, allogeneic bone graft is preferred. The bone strength of the defect area would not be affected after union. Meanwhile, it can also avoid the trauma and prolonged surgery time of the autograft [8].

## 5. Conclusions

Compared with conventional arthroscopy, the application of 3D printing technology in treating the bone cyst of the foot and ankle can help the surgeon design a preoperative plan and perform rehearsals in advance. It makes it easier for an inexperienced surgeon to understand the anatomical structure and facilitate a faster establishment of the arthroscopic portal. There are still limitations to this study. The follow-up period is relatively short with only 12 months, and the long-term efficacy of the patient has not been followed. In addition, due to the small number of cases of bone cysts in the foot and ankle, as well as the different locations of bone cysts in each group, the number of patients included in this study is also quite small. Thus, it is difficult to analyze the influence of different bone grafting methods or explore the perioperative complications. Future multicenter case-control studies need to be conducted to overcome these deficiencies.

## Figures and Tables

**Table 1:** Patient data.

	Experimental group (*n* = 11)	Control group (*n* = 10)	*P*
Sex (*n*)		
Female	3	7	0.086
Male	8	3	
Age (yr)	39.5 ± 5.8	45.0 ± 5.2	0.485
Location of cyst		
Talus	6	5	0.484
Distal tibia	3	4	
Calcaneus	1	2	
Preoperative symptom duration (mo)	21.0 ± 6.1	34.7 ± 13.1	0.340
Pain (*n*)	11	10	NA

Abbreviations: NA: not applicable.

**Table 2:** The two groups' comparative follow-up study.

Groups	Experimental group	Control group	*t*-test
Cases (*n*)	11	10	
Preoperative AOFAS scores (pt)	59.5 ± 2.1	61.2 ± 2.4	*P* = 0.587
AOFAS scores at the last follow-up (pt)	94.7 ± 0.7	92.5 ± 1.3	*P* = 0.135
Preoperative VAS scores (pt)	7.2 ± 0.3	6.9 ± 0.3	*P* = 0.522
VAS scores at the last follow-up (pt)	0.4 ± 0.2	0.5 ± 0.2	*P* = 0.614
Time of establishing surgical approach (seconds)	204.5 ± 6.5	318.1 ± 7.4	*P* = 0.000
Intraoperative fluoroscopy times	2.2 ± 0.1	3.7 ± 0.2	*P* = 0.000
Intraoperative blood loss	14.5 ± 1.1	30.5 ± 5.8	*P* = 0.009

Abbreviations: AOFAS: American Orthopaedic Foot and Ankle Society; VAS: visual analog scale.

## Data Availability

The corresponding author Xiaojun Duan can make data available upon request, email: duanxiaojun@hotmail.com.
